# Fetal Superior Vena Cava Blood Flow and Its Fraction of Cardiac Output: A Longitudinal Ultrasound Study in the Second Half of Pregnancy

**DOI:** 10.3389/fped.2021.658502

**Published:** 2021-07-06

**Authors:** Maria Stefopoulou, Jonas Johnson, Lotta Herling, Peter Lindgren, Torvid Kiserud, Ganesh Acharya

**Affiliations:** ^1^Division of Obstetrics and Gynecology, Department of Clinical Science, Intervention & Technology (CLINTEC), Karolinska Institutet, Stockholm, Sweden; ^2^Center for Fetal Medicine Karolinska, University Hospital, Stockholm, Sweden; ^3^Women's Health and Perinatology Research Group, Department of Clinical Medicine, Faculty of Health Sciences, UiT-The Arctic University of Norway and Department of Obstetrics and Gynecology, University Hospital of North Norway, Tromsø, Norway; ^4^Department of Clinical Science, University of Bergen, Bergen, Norway; ^5^Department of Obstetrics and Gynecology, Haukeland University Hospital, Bergen, Norway

**Keywords:** blood flow, fetal hemodynamics, fetal brain, cerebral circulation, superior vena cava, Doppler

## Abstract

**Introduction:** In the fetus, a large proportion of the superior vena cava blood flow (Q_SVC_) comes from the brain. To provide the possibility of using this blood flow as a representation of fetal brain circulation, we aimed to determine the fetal Q_SVC_ and its fraction of cardiac output during the second half of physiological pregnancies.

**Materials and Methods:** This was a prospective longitudinal study specifically designed for studying fetal hemodynamic development. Healthy women with singleton low-risk pregnancies were included. Ultrasonography was performed at 4-weekly intervals from 20^+0^ gestational weeks to term. Doppler velocity recordings of the superior vena cava (SVC) and cardiac ventricular outflow tracts were used to obtain the time-averaged maximum velocities (TAMxV). Vessel diameters were measured to calculate their cross-sectional areas (CSA): π(diameter/2)^2^. Blood flow (Q) was computed as: h^*^TAMxV^*^CSA, *h* being the spatial blood velocity profile, to obtain Q_SVC_ and cardiac outputs. The sum of left and right ventricular cardiac outputs constituted the combined cardiac output (CCO). Ultrasound biometry based estimated fetal weight and brain weight were used to normalize the flow. Q_SVC_ was also expressed as the fraction (%) of CCO. Gestational age specific percentiles were established for each blood flow parameter using multilevel modeling.

**Results:** Totally, 134 of the 142 included women were eligible for the study with 575 sets of observations. The SVC mean diameter (19–52 mm), mean TAMxV (8.83–16.14 cm/s), and Q_SVC_ (15.4–192.0 ml/min) increased significantly during the second half of pregnancy (*p* < 0.001) while the mean Q_SVC_ normalized by estimated fetal weight (49 ml/min/kg) and by estimated brain weight (50 ml/min/100 g) were relatively stable. Similarly, the mean CCO increased (156–1,776 ml/min; *p* < 0.001) while the normalized CCO (509 ± 13 ml/min/kg) and Q_SVC_ as a fraction of CCO (10 ± 0.92%) did not change significantly with gestational age.

**Conclusion:** We provide reference values for fetal Q_SVC_ which increases significantly with gestation, and constitutes roughly 10% of the fetal CCO at any time during the second half of pregnancy.

## Introduction

Blood supply to the brain during intrauterine life is physiologically interesting and clinically relevant. Several adverse perinatal events are associated with abnormal cerebral blood flow, but its direct measurement is difficult in the perinatal period, so alternative representations have been introduced. The brain has a well-developed autoregulation of perfusion, and since variation in impedance is reflected in the waveform of the middle cerebral artery (MCA) blood velocity, this Doppler assessment has become a valuable clinical tool for monitoring fetal well-being, identifying circulatory compromise, predicting perinatal outcomes and guiding treatment strategies (1). A further extension of the technique utilizes the ratio between umbilical artery (UA) and MCA pulsatility index (PI) to reflect stages of fetal “brain sparing” during intrauterine hypoxemia (2, 3), although with some limitations (4). Doppler velocimetry also determines a raised peak blood velocity in the MCA as a sign of fetal anemia (5, 6). However useful these components of fetal cerebral hemodynamics are, we believe that measuring the volume of blood flow through the brain would add an important physiological parameter to clinical diagnosis and management.

Determining the fetal cerebral blood supply non-invasively by measuring blood flow in the carotid and vertebral arteries has not been feasible in human pregnancies. In the neonatal period, the measurement of blood flow in the superior vena cava (SVC) has turned out to be a useful alternative representation of the cerebral circulation in clinical medicine (7). And, the same ultrasound technique has successfully been tested out prenatally (8). The question is, how well the SVC represents the cerebral circulation, as it also drains venous return from the upper body. Experimental studies in primate fetuses near term have reported SVC blood flow to constitute 23% of the combined cardiac output (CCO) and brain blood flow 15.7% of CCO (9). An early study on exteriorized previable human fetuses found similarly that roughly 25% of the CCO was flowing in the SVC at mid-gestation, and that the brain circulated 14% (10). Also, fetal lamb studies report similar results with 21% of the CCO received through the SVC, but the brain contribution is lower as sheep brain is considerably smaller than in humans (2–3 vs. 13% of body weight) (11, 12).

Based on this background, we assumed that SVC blood flow, to a large extent, represents brain circulation in the human fetus and aimed at determining fetal SVC blood flow and its fraction of CCO during the second half of healthy pregnancies in order to establish normative data including reference ranges.

## Materials and Methods

This prospective observational study was embedded in a larger project investigating physiological changes in fetal cardiovascular function during the second half of pregnancy. The study had a longitudinal design including low-risk singleton pregnancies for serial ultrasound scans at ~4-weekly intervals during 20–41 weeks of gestation. Normative data on blood flow-based cerebroplacental ratio (Q-CPR), umbilicocerebral ratio (Q-UCR), and SVC velocities and pulsatility index for vein from this study population have been published previously (13, 14). Here we address absolute and weight-indexed Q_SVC_ and its fraction of CCO as a representation of fetal brachio-cephalic circulation.

The study took place at the University Hospital of North Norway, Tromsø, Norway. Written information about the study was sent to women with the appointment letter to attend the routine second trimester ultrasound screening. During that session, the women were assessed for eligibility and those who consented in written, were consecutively enrolled during February 2009 to December 2012. Inclusion criteria were, woman's age ≥18 years, singleton pregnancy, live fetus, gestation ≥18 weeks and <24 weeks confirmed by ultrasound biometry, and absence of any major congenital fetal or placental abnormality. Women with a previous obstetric history of preeclampsia, gestational diabetes or preterm labor before 34 weeks of gestation, and those with illness known to affect the course and outcome of pregnancy (such as chronic hypertension, renal disease, autoimmune disease, pre-gestational diabetes mellitus) were excluded. Once included, none were excluded due to complications during pregnancy.

An ultrasound examination was performed by a specialist obstetrician with at least 3 years of fetal ultrasound scanning experience. The same Vivid 7 Dimension ultrasound machine (GE Vingmed Ultrasound AS, Horten, Norway) with a 4MS (1.5–4.3 MHz) sector transducer was used for all measurements. Fetal weight was estimated by ultrasound biometry of head, abdomen and femur using Hadlock formula 3 (15). Fetal Brain weight at 28–40 weeks of gestation was estimated based on an equation proposed by Dobbing and Sands that has been validated for the third trimester (16): Fetal brain weight (g) = (Head circumference, cm)^3^/*a* −*b*/2^*^(Head circumference, cm); where the coefficients *a* = 100 and *b* = 3,000. The brains for this validation were obtained at necropsy from 36 human fetuses demised between 25 weeks and term gestation. These fetuses were selected to represent normal fetuses on the basis of having normal growth (body weight being within 1SD for the gestational age) and no neuropathology (17).

**Figure 1:**
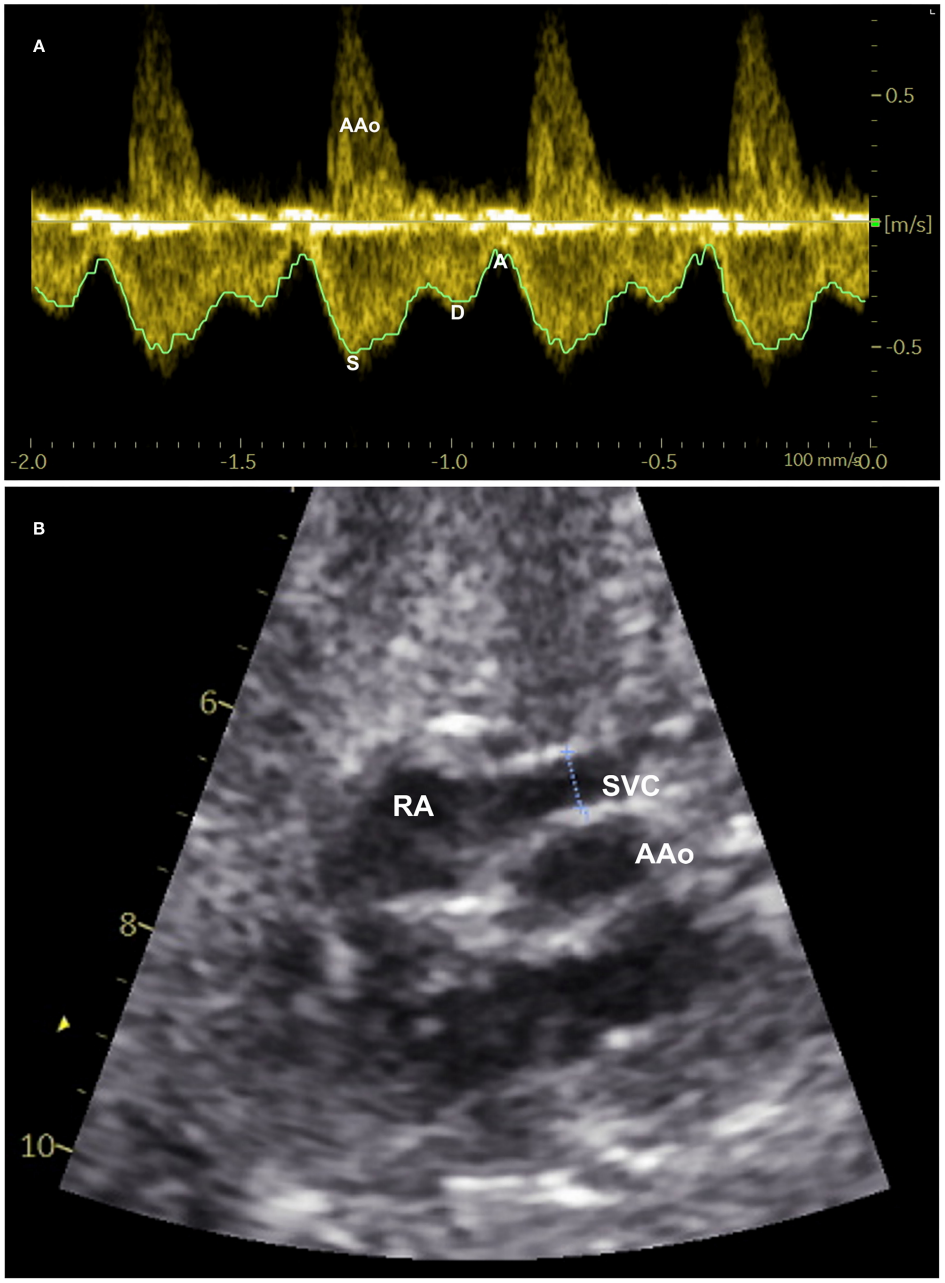
Measurement of superior vena cava (SVC) blood flow: **(A)** Spectral pulsed-wave Doppler velocity waveforms of the fetal SVC with tracing of maxium velocity envelope over cardiac cycles to measure time-averaged maximum velocity. S-maximum velocity during systole, D-peak velocity during diastole, and A-minimum velocity during atrial contraction. **(B)** Two-dimensional B-mode ultrasound image of SVC demonstrating measurement of its inner diameter (dotted line between two calipers) shortly above the entrance to the right atrium (RA). AAo-Ascending aorta.

To measure SVC blood flow, the blood velocity waveforms were recorded from the SVC using pulsed-wave Doppler (8) (Figure 1A) and its inner diameter was determined using two-dimensional B-mode ultrasound (18) (Figure 1B) as described previously. An average value of three measurements was used for the calculation of blood flow. To measure the cardiac outputs, blood flow velocity waveforms were obtained from the left and right ventricular outflow tracts and their respective diameters were measured at the level of aortic and pulmonary valves using the leading-edge technique (19). Left and right ventricular outputs constituted the combined cardiac output (CCO).

From the blood flow velocity waveforms of each blood vessel, the time-averaged maximum velocity (TAMxV) was measured. An average value of three cardiac cycles was recorded. An average of three diameter measurements were used for computing the cross-sectional areas (CSA) of the vessel. The vessel CSA was determined as: π(diameter/2)^2^. Blood flow volume (Q) of each vessel was calculated as: h^*^TAMxV^*^CSA, where *h* represents the spatial blood velocity profile of the vessel. For the aorta and pulmonary artery, the spatial velocity profile was considered to be flat (*h* = 1) (19–21), and for the SVC, partially blunted (*h* = 0.7) (8). Normalized (weight-indexed) Q was calculated as Q/estimated fetal weight. The Q_SVC_ was also normalized by fetal head circumference and estimated fetal brain weight. The SVC fraction of fetal CCO was calculated as: (Q_SVC_/CCO)^*^100.

All women continued to attend their routine antenatal care in parallel with the study. Delivery and neonatal outcomes were obtained from the electronic medical records.

### Statistical Analyses

Data analysis was performed using IBM SPSS Statistics for Windows, Version 24.0. (IBM Corp, Armonk, NY) and MATLAB R2019a (Matworks, Inc. Natick, MA). Distribution of data was checked for each variable. A Box-Cox transformation lambda value (λ) was calculated for each response (dependent) variable, and rounded to the nearest integer. Based on this value all the dependent variables were transformed to achieve best possible normal distribution, as follows: λ = 1: no transformation; λ = 0: logarithmic transformation; λ = 0.5; square root transformation.

The best fitting fractional polynomials were chosen from a list of 44 regression models based on R^2^ value to construct mean curves of each dependent variable in relation to gestational age. The mean and percentiles for each gestational week were calculated using multilevel modeling accounting for the repeated measures design (22). The variance between measurements within the same fetus was the first level and the variance between participating pregnant women as the second level. The 2.5, 5, and 10th percentiles were calculated by subtracting 1.960 standard deviation (SD), 1.645 SD, and 1.282 SD from the mean, respectively and the 97.5, 90, and 75th percentiles by adding similar SD values, respectively. The 95% confidence intervals were calculated for the 5, 50, 95th percentiles.

Since normal distribution of the variables was achieved by appropriate transformation of the primary measurements and the reference intervals were initially calculated on the transformed scale, reverse transformations were performed to obtain the values of gestational-age specific reference percentiles in the primary measurement scales. Associations between each variable and the gestational age was tested using the mean vector for each variable from the mixed models accounting for longitudinal design. *P* < 0.05 was considered significant.

### Ethics Approval

The study was approved by the Regional Committee for Medical and Health Research Ethics –North Norway (REK Nord 105/2008) and an informed written consent was obtained from the participants at inclusion.

## Results

Of the 142 pregnant women enrolled to the study, eight were excluded due to missing data leaving 134 with 575 observations. Adequate quality images available were used for measuring blood velocities and diameters of the SVC (490 paired sets of recordings), and the left and right cardiac ventricular outflow tracts (542 and 524 paired sets of recordings, respectively), and data were entered for statistical analysis. Demographic characteristics and pregnancy outcomes have been reported previously (13). In brief, the median age was 30 years (range, 19-39), 45.5% were nullipara, 4.4% developed pregnancy complications (4 preeclampsia, 1 gestational diabetes and 1 abruptio placentae), 3.7% delivered preterm, and 12.7% were delivered by cesarean section. Mean birthweight was 3,600 g (range, 2,252–4,636 g), 1.4% (*n* = 2) had a 5-minute Apgar score <7, and there was no perinatal death.

**Figure 2:**
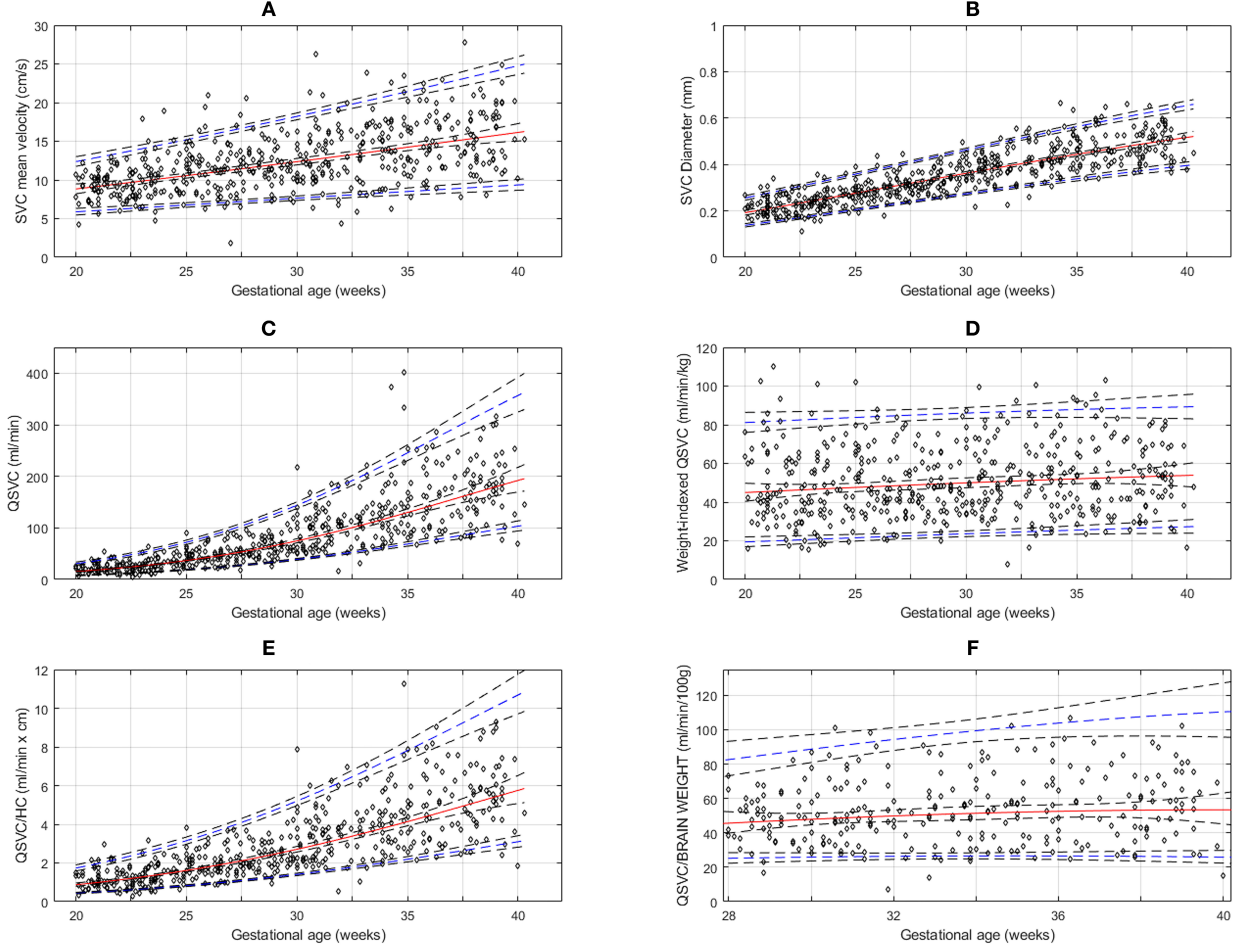
Reference charts for fetal superior vena cava (SVC): **(A)** mean velocity, **(B)** diameter, **(C)** volume blood flow (Q_SVC_), **(D)** weight-indexed Q_SVC_, **(E)** volume blood flow normalized by head circumference (Qsvc/HC) at 20-40 weeks of gestation, and **(F)** volume blood flow (Q_SVC_) normalized by estimated fetal brain weight at 28–40 weeks of gestation. The solid red line represents the 50th percentile, the blue lines represent the 5 and 95th percentiles and the interrupted black lines represent their respective 95% confidence limits.

The gestational age specific reference percentiles of SVC mean velocity, SVC diameter, Q_SVC_, and Q_SVC_ normalized by estimated fetal weight, fetal head circumference, and by fetal brain weight are presented in Tables 1–6. The SVC mean velocity (8.83 to 16.14 cm/s), mean diameter (19 to 52 mm), Q_SVC_ (5.4 to 192.0 ml/min) and Q_SVC_ normalized by fetal head circumference (0.87 to 5.69 ml/cm) increased (*p* < 0.001) during the second half of pregnancy (Figures 2A–D). However, the weight-indexed Q_SVC_ (mean, 49.2 ml/min/kg) remained relatively stable ranging from 45.6 to 53.2 ml/min/kg during 20–40 weeks of pregnancy (Figure 2E). The Q_SVC_ normalized by estimated fetal brain weight (mean, 50.42 ml/min/100 g) did not change significantly ranging from 45.9 to 53.8 during 28–40 weeks (Figure 2F).

**Table 1:** Percentiles of fetal superior vena cava mean blood velocity (cm/s) at 20–40 weeks of gestational age (GA).

		**Percentile**						
**GA (weeks)**	***n***	**2.5th**	**5th**	**10th**	**50th**	**90th**	**95th**	**97.5th**
20	21	5.39	5.88	6.48	8.83	11.54	12.37	13.12
21	33	5.54	6.06	6.69	9.17	12.04	12.92	13.71
22	30	5.69	6.23	6.90	9.51	12.54	13.48	14.32
23	32	5.83	6.41	7.11	9.86	13.06	14.04	14.93
24	17	5.98	6.59	7.32	10.21	13.58	14.62	15.55
25	30	6.13	6.76	7.53	10.56	14.10	15.20	16.18
26	29	6.28	6.94	7.74	10.91	14.64	15.79	16.82
27	28	6.42	7.11	7.95	11.27	15.18	16.39	17.47
28	27	6.57	7.29	8.16	11.63	15.72	16.99	18.13
29	21	6.72	7.46	8.37	12.00	16.27	17.60	18.80
30	34	6.86	7.64	8.58	12.36	16.83	18.22	19.47
31	27	7.01	7.81	8.80	12.73	17.40	18.85	20.16
32	18	7.15	7.99	9.01	13.10	17.97	19.48	20.85
33	25	7.29	8.16	9.22	13.48	18.54	20.12	21.54
34	25	7.44	8.33	9.43	13.85	19.12	20.77	22.25
35	27	7.58	8.51	9.64	14.23	19.71	21.42	22.96
36	20	7.72	8.68	9.85	14.61	20.30	22.08	23.68
37	20	7.86	8.85	10.06	14.99	20.89	22.74	24.41
38	25	7.99	9.02	10.27	15.37	21.49	23.41	25.14
39	18	8.13	9.19	10.48	15.76	22.10	24.09	25.88
40	2	8.26	9.35	10.69	16.14	22.71	24.77	26.63

**Table 2:** Percentiles of fetal superior vena cava diameter (cm) at 20–40 weeks of gestational age (GA).

		**Percentile**						
**GA (weeks)**	***n***	**2.5th**	**5th**	**10th**	**50th**	**90th**	**95th**	**97.5th**
20	20	0.13	0.14	0.15	0.19	0.24	0.26	0.27
21	32	0.14	0.15	0.16	0.21	0.26	0.28	0.29
22	29	0.15	0.16	0.18	0.23	0.28	0.30	0.31
23	32	0.17	0.18	0.19	0.24	0.30	0.32	0.33
24	17	0.18	0.19	0.21	0.26	0.32	0.34	0.35
25	28	0.19	0.21	0.22	0.28	0.34	0.36	0.38
26	28	0.21	0.22	0.23	0.29	0.36	0.38	0.40
27	27	0.22	0.23	0.25	0.31	0.38	0.40	0.42
28	27	0.23	0.25	0.26	0.33	0.40	0.42	0.44
29	21	0.25	0.26	0.28	0.35	0.42	0.44	0.46
30	34	0.26	0.27	0.29	0.36	0.44	0.46	0.48
31	25	0.27	0.29	0.31	0.38	0.46	0.48	0.50
32	18	0.28	0.30	0.32	0.40	0.48	0.50	0.53
33	24	0.30	0.31	0.33	0.41	0.50	0.52	0.55
34	25	0.31	0.33	0.35	0.43	0.52	0.54	0.57
35	25	0.32	0.34	0.36	0.44	0.54	0.56	0.59
36	18	0.33	0.35	0.37	0.46	0.55	0.58	0.61
37	19	0.34	0.36	0.39	0.47	0.57	0.60	0.63
38	24	0.35	0.37	0.40	0.49	0.59	0.62	0.65
39	17	0.36	0.38	0.41	0.50	0.61	0.64	0.66
40	2	0.37	0.40	0.42	0.52	0.62	0.65	0.68

**Table 3:** Percentiles fetal superior vena cava volume blood flow (ml/min) at 20–40 weeks of gestational age (GA).

		**Percentile**						
**GA (weeks)**	***n***	**2.5th**	**5th**	**10th**	**50th**	**90th**	**95th**	**97.5th**
20	20	6.79	7.75	9.02	15.42	26.38	30.70	35.03
21	32	8.22	9.37	10.90	18.59	31.70	36.88	42.04
22	29	9.88	11.26	13.09	22.26	37.86	44.01	50.14
23	32	11.81	13.44	15.61	26.49	44.93	52.18	59.42
24	17	14.02	15.95	18.51	31.31	52.98	61.48	69.96
25	28	16.53	18.80	21.80	36.79	62.07	71.99	81.86
26	28	19.38	22.02	25.52	42.95	72.27	83.74	95.17
27	27	22.57	25.64	29.68	49.82	83.61	96.81	109.94
28	27	26.12	29.65	34.30	57.42	96.10	111.19	126.20
29	21	30.04	34.07	39.39	65.76	109.77	126.91	143.94
30	34	34.33	38.91	44.95	74.83	124.58	143.93	163.13
31	25	38.98	44.15	50.96	84.62	140.50	162.19	183.71
32	17	43.97	49.78	57.42	95.08	157.44	181.61	205.57
33	24	49.30	55.76	64.28	106.15	175.31	202.07	228.58
34	25	54.91	62.08	71.50	117.76	193.96	223.40	252.54
35	25	60.78	68.67	79.03	129.81	213.24	245.41	277.24
36	17	66.85	75.47	86.79	142.19	232.94	267.89	302.43
37	19	73.06	82.42	94.72	154.76	252.85	290.56	327.82
38	24	79.33	89.45	102.71	167.37	272.72	313.16	353.07
39	17	85.60	96.45	110.67	179.85	292.28	335.37	377.86
40	2	91.78	103.35	118.49	192.04	311.26	356.87	401.83

**Table 4:** Percentiles of weight-indexed fetal superior vena cava volume blood flow (ml/min/kg) at 20-40 weeks of gestational age (GA).

		**Percentile**						
**GA (weeks)**	***n***	**2.5th**	**5th**	**10th**	**50th**	**90th**	**95th**	**97.5th**
20	20	15.70	19.38	24.11	44.95	72.22	81.11	89.24
21	32	16.11	19.83	24.59	45.50	72.79	81.67	89.80
22	29	16.52	20.27	25.06	46.04	73.34	82.22	90.34
23	32	16.93	20.71	25.52	46.56	73.88	82.76	90.87
24	17	17.34	21.14	25.98	47.08	74.41	83.28	91.38
25	28	17.74	21.57	26.44	47.59	74.92	83.78	91.87
26	28	18.14	21.99	26.88	48.09	75.41	84.26	92.34
27	26	18.54	22.41	27.32	48.57	75.89	84.73	92.80
28	27	18.93	22.82	27.76	49.05	76.36	85.18	93.24
29	21	19.32	23.23	28.18	49.51	76.81	85.62	93.66
30	34	19.70	23.63	28.60	49.96	77.24	86.04	94.06
31	25	20.08	24.02	29.01	50.40	77.66	86.44	94.44
32	17	20.45	24.41	29.42	50.83	78.06	86.82	94.81
33	24	20.81	24.80	29.82	51.24	78.44	87.19	95.16
34	25	21.17	25.17	30.20	51.65	78.81	87.54	95.49
35	24	21.53	25.54	30.58	52.04	79.16	87.87	95.80
36	17	21.88	25.90	30.96	52.42	79.50	88.18	96.09
37	17	22.22	26.25	31.32	52.78	79.81	88.48	96.36
38	23	22.55	26.60	31.67	53.13	80.12	88.76	96.62
39	17	22.88	26.94	32.02	53.47	80.40	89.02	96.85
40	2	23.20	27.27	32.35	53.80	80.67	89.26	97.07

**Table 5:** Percentiles of fetal superior vena cava volume blood flow normalized by head circumference (ml/min/cm) at 20–40 weeks of gestational age (GA).

		**Percentile**						
**GA (weeks)**	***n***	**2.5th**	**5th**	**10th**	**50th**	**90th**	**95th**	**97.5th**
20	20	0.39	0.44	0.51	0.87	1.49	1.74	1.98
21	32	0.44	0.50	0.58	1.00	1.70	1.97	2.25
22	29	0.50	0.57	0.66	1.13	1.92	2.23	2.54
23	32	0.57	0.65	0.75	1.28	2.16	2.51	2.86
24	17	0.64	0.73	0.85	1.44	2.43	2.82	3.21
25	28	0.73	0.82	0.96	1.61	2.72	3.15	3.58
26	28	0.81	0.92	1.07	1.80	3.03	3.51	3.99
27	26	0.91	1.03	1.20	2.01	3.36	3.89	4.42
28	27	1.01	1.15	1.33	2.23	3.72	4.30	4.88
29	21	1.12	1.28	1.47	2.46	4.10	4.74	5.38
30	34	1.24	1.41	1.63	2.71	4.50	5.20	5.89
31	25	1.37	1.55	1.79	2.97	4.93	5.69	6.44
32	17	1.50	1.70	1.96	3.24	5.37	6.19	7.01
33	24	1.64	1.86	2.14	3.53	5.83	6.72	7.60
34	25	1.79	2.02	2.33	3.83	6.30	7.26	8.20
35	24	1.94	2.19	2.52	4.14	6.79	7.81	8.83
36	17	2.10	2.36	2.72	4.45	7.29	8.38	9.46
37	18	2.26	2.54	2.92	4.77	7.80	8.96	10.10
38	22	2.42	2.73	3.13	5.10	8.31	9.53	10.75
39	17	2.58	2.91	3.34	5.43	8.81	10.11	11.39
40	2	2.75	3.10	3.55	5.75	9.32	10.68	12.03

**Table 6:** Percentiles of fetal superior vena cava volume blood flow normalized by brain weight (ml/min/100 g) at 28–40 weeks of gestational age (GA).

		**Percentile**						
**GA (weeks)**	***n***	**2.5th**	**5th**	**10th**	**50th**	**90th**	**95th**	**97.5th**
20								
21								
22								
23								
24								
25								
26								
27								
28	27	22.50	25.21	28.74	45.64	72.49	82.63	92.58
29	21	22.78	25.58	29.23	46.82	74.99	85.69	96.21
30	34	23.01	25.89	29.65	47.91	77.41	88.67	99.77
31	25	23.19	26.15	30.02	48.92	79.73	91.55	103.22
32	17	23.32	26.35	30.33	49.85	81.93	94.31	106.56
33	24	23.39	26.49	30.57	50.67	84.01	96.94	109.76
34	25	23.42	26.57	30.74	51.40	85.94	99.41	112.80
35	24	23.39	26.60	30.84	52.02	87.73	101.72	115.67
36	17	23.31	26.56	30.88	52.53	89.35	103.86	118.34
37	18	23.18	26.47	30.84	52.92	90.80	105.80	120.81
38	22	23.00	26.32	30.74	53.20	92.07	107.54	123.05
39	17	22.77	26.11	30.57	53.36	93.14	109.06	125.06
40	2	22.49	25.84	30.33	53.40	94.02	110.35	126.81

*No data presented for 20–27 weeks as the formula used for brain weight estimation is not valid before 28 weeks of gestation*.

**Figure 3:**
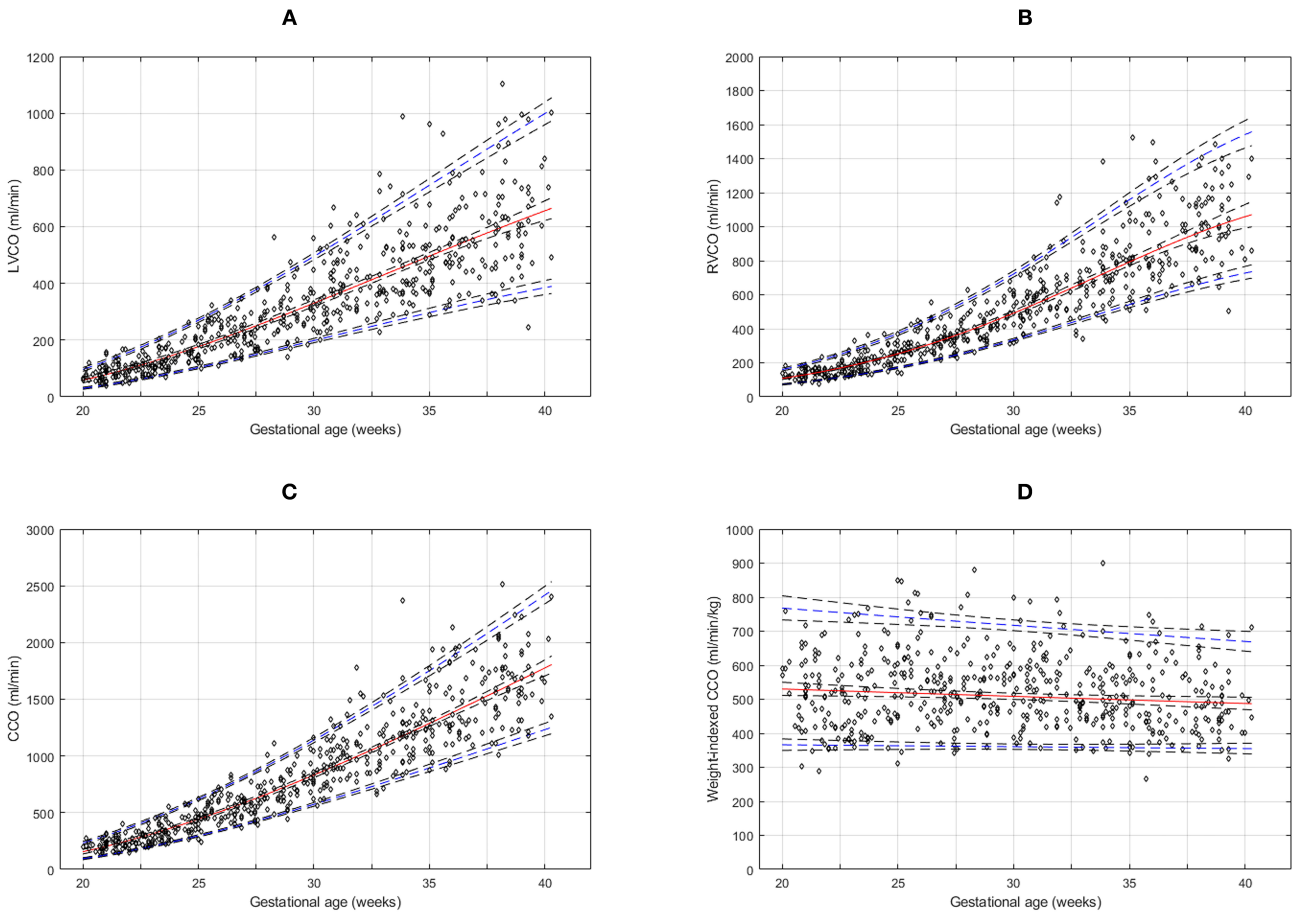
Reference charts for fetal cardiac ventricular outputs: **(A)** left ventricular cardiac output (LVCO), **(B)** right ventricular cardiac output (RVCO), **(C)** combined ventricular cardiac output (CCO), and **(D)** normalized (weight-indexed) CCO at 20–40 weeks of gestation. The solid red line represents the 50th percentile, the blue lines represent the 5 and 95th percentiles and the interrupted black lines represent their respective 95% confidence limits.

**Figure 4:**
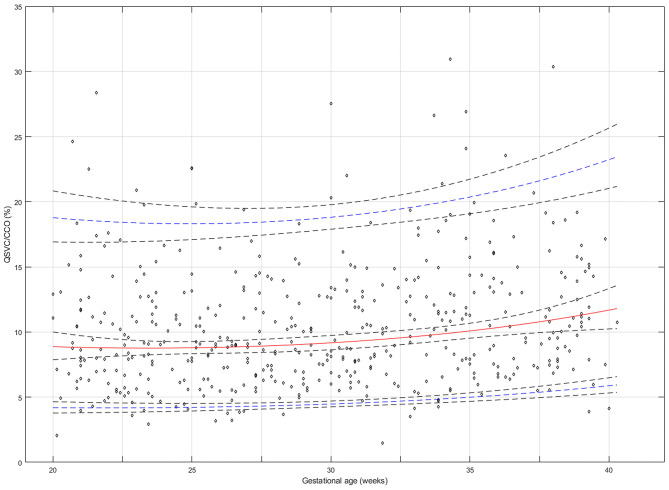
Reference chart for the superior vena cava (SVC) fraction (%) of fetal combined cardiac output (CCO) distributed to the brain and upper body at 20–40 weeks of gestation. The solid red line represents the 50th percentile, the blue lines represent the 5 and 95th percentiles and the interrupted black lines represent their respective 95% confidence limits.

The reference percentiles for left and right ventricular output, CCO and weight-indexed CCO are presented in Tables 7–10. Reference charts fitted with 5, 50, and 95th percentiles and their corresponding 95% CI are shown in Figures 3A–D. The mean CCO increased from 156 ml/min at 20 weeks to 1,776 ml/min at 40 weeks (*p* < 0.001), whereas the weight-indexed CCO (mean, 509 (SD,13) ml/min/kg; range, 488 to 530 ml/min/kg) did not change significantly with gestational age (*p* = 0.07). The Q_SVC_ as the fraction of fetal CCO was 10% (SD,0.92) and hardly changed during the second half of pregnancy (Table 11; Figure 4).

**Table 7:** Percentiles of left ventricular cardiac output (ml/min) at 20–40 weeks of gestational age (GA).

		**Percentile**						
**GA (weeks)**	***n***	**2.5th**	**5th**	**10th**	**50th**	**90th**	**95th**	**97.5th**
20	21	25.68	30.07	35.56	58.62	87.43	96.63	104.98
21	34	36.34	42.01	49.04	78.25	114.24	125.67	136.02
22	30	48.43	55.48	64.20	100.09	143.90	157.75	170.28
23	35	61.77	70.32	80.85	123.92	176.16	192.62	207.49
24	20	76.20	86.33	98.80	149.56	210.79	230.04	247.42
25	31	91.55	103.37	117.89	176.78	247.56	269.77	289.81
26	29	107.66	121.26	137.93	205.40	286.25	311.58	334.43
27	30	124.40	139.85	158.78	235.22	326.64	355.24	381.04
28	27	141.62	159.00	180.27	266.06	368.51	400.54	429.42
29	21	159.18	178.55	202.25	297.75	411.66	447.26	479.35
30	38	176.95	198.37	224.57	330.10	455.89	495.19	530.61
31	28	194.80	218.33	247.10	362.95	501.01	544.13	582.99
32	21	212.62	238.29	269.69	396.14	546.81	593.88	636.30
33	27	230.28	258.14	292.23	429.50	593.13	644.24	690.32
34	27	247.68	277.77	314.58	462.90	639.77	695.04	744.86
35	30	264.72	297.05	336.62	496.17	686.58	746.10	799.75
36	24	281.29	315.89	358.26	529.20	733.37	797.22	854.79
37	19	297.31	334.19	379.37	561.83	780.00	848.26	909.81
38	28	312.69	351.86	399.86	593.94	826.29	899.04	964.65
39	18	327.34	368.80	419.63	625.41	872.11	949.40	1019.13
40	4	341.20	384.93	438.59	656.12	917.31	999.20	1073.10

**Table 8:** Percentiles of right ventricular cardiac output (ml/min) at 20–40 weeks of gestational age (GA).

		**Percentile**						
**GA (weeks)**	***n***	**2.5th**	**5th**	**10th**	**50th**	**90th**	**95th**	**97.5th**
20	17	67.61	72.89	79.49	107.95	146.61	159.88	172.37
21	34	81.47	87.82	95.75	129.94	176.35	192.28	207.27
22	27	97.43	105.00	114.47	155.24	210.55	229.52	247.37
23	34	115.65	124.62	135.82	184.09	249.50	271.93	293.02
24	20	136.25	146.79	159.96	216.65	293.44	319.76	344.51
25	30	159.32	171.62	186.98	253.08	342.55	373.20	402.02
26	29	184.91	199.15	216.93	293.42	396.88	432.32	465.62
27	30	213.00	229.37	249.80	337.65	456.40	497.05	535.26
28	25	243.53	262.20	285.50	385.65	520.92	567.22	610.71
29	20	276.35	297.49	323.87	437.18	590.13	642.45	691.60
30	37	311.25	335.01	364.64	491.89	663.53	722.23	777.35
31	28	347.95	374.44	407.48	549.31	740.50	805.84	867.20
32	20	386.06	415.39	451.96	608.85	820.21	892.42	960.21
33	26	425.15	457.37	497.54	669.81	901.72	980.92	1055.26
34	27	464.69	499.83	543.63	731.36	983.92	1070.14	1151.05
35	30	504.13	542.15	589.55	792.60	1065.60	1158.75	1246.16
36	22	542.82	583.67	634.57	852.56	1145.43	1245.33	1339.04
37	19	580.11	623.67	677.93	910.20	1222.05	1328.37	1428.10
38	27	615.34	661.43	718.84	964.48	1294.06	1406.37	1511.71
39	18	647.83	696.24	756.52	1014.36	1360.07	1477.83	1588.26
40	4	676.94	727.40	790.24	1058.85	1418.76	1541.32	1656.21

**Table 9:** Percentiles of combined ventricular cardiac output (ml/min) at 20-40 weeks of gestational age (GA).

		**Percentile**						
**GA (weeks)**	***n***	**2.5th**	**5th**	**10th**	**50th**	**90th**	**95th**	**97.5th**
20	17	83.93	93.97	106.25	155.65	214.45	232.81	249.35
21	34	114.12	126.70	142.02	203.09	275.06	297.41	317.52
22	27	148.20	163.53	182.13	255.85	342.06	368.74	392.70
23	34	185.88	204.17	226.31	313.60	415.11	446.43	474.53
24	20	226.90	248.35	274.25	376.04	493.85	530.12	562.64
25	30	271.00	295.79	325.70	442.84	577.96	619.48	656.67
26	28	317.92	346.23	380.36	513.72	667.08	714.14	756.28
27	30	367.40	399.42	437.98	588.37	760.91	813.79	861.12
28	25	419.20	455.10	498.29	666.49	859.10	918.08	970.84
29	19	473.09	513.01	561.03	747.80	961.35	1026.69	1085.12
30	37	528.81	572.93	625.96	832.02	1067.34	1139.29	1203.63
31	28	586.16	634.61	692.82	918.87	1176.77	1255.58	1326.05
32	20	644.89	697.81	761.39	1008.08	1289.33	1375.25	1452.05
33	26	704.80	762.33	831.41	1099.39	1404.73	1497.98	1581.33
34	27	765.67	827.92	902.68	1192.53	1522.68	1623.48	1713.58
35	30	827.30	894.39	974.95	1287.26	1642.90	1751.47	1848.50
36	22	889.48	961.52	1048.02	1383.33	1765.11	1881.65	1985.79
37	18	952.01	1029.11	1121.68	1480.49	1889.03	2013.73	2125.18
38	26	1014.72	1096.96	1195.71	1578.52	2014.40	2147.46	2266.37
39	18	1077.41	1164.89	1269.92	1677.17	2140.95	2282.55	2409.09
40	4	1139.91	1232.69	1344.12	1776.22	2268.44	2418.74	2553.07

**Table 10:** Percentiles of normalized (weight-indexed) combined ventricular cardiac output (ml/min/kg) at 20–40 weeks of gestational age.

		**Percentile**						
**GA (weeks)**	***n***	**2.5th**	**5th**	**10th**	**50th**	**90th**	**95th**	**97.5th**
20	16	341.09	366.15	397.32	530.23	707.59	767.83	824.25
21	34	340.73	365.58	396.47	528.01	703.19	762.61	818.24
22	27	340.37	365.01	395.63	525.80	698.82	757.43	812.26
23	33	340.01	364.44	394.78	523.61	694.47	752.28	806.34
24	20	339.65	363.88	393.94	521.42	690.15	747.17	800.45
25	30	339.30	363.31	393.10	519.24	685.85	742.09	794.61
26	28	338.94	362.74	392.26	517.07	681.59	737.04	788.81
27	30	338.58	362.18	391.42	514.91	677.35	732.03	783.05
28	25	338.23	361.62	390.58	512.75	673.13	727.06	777.34
29	19	337.87	361.05	389.75	510.61	668.95	722.11	771.66
30	37	337.51	360.49	388.92	508.47	664.78	717.20	766.03
31	28	337.16	359.93	388.09	506.35	660.65	712.33	760.44
32	20	336.80	359.37	387.26	504.23	656.54	707.49	754.89
33	26	336.45	358.81	386.43	502.12	652.45	702.68	749.38
34	27	336.10	358.25	385.61	500.03	648.40	697.90	743.91
35	29	335.74	357.70	384.78	497.94	644.36	693.16	738.48
36	22	335.39	357.14	383.96	495.85	640.35	688.44	733.09
37	16	335.04	356.59	383.14	493.78	636.37	683.76	727.74
38	25	334.68	356.03	382.32	491.72	632.41	679.12	722.43
39	18	334.33	355.48	381.51	489.66	628.48	674.50	717.16
40	4	333.98	354.92	380.69	487.61	624.57	669.91	711.92

**Table 11:** Percentiles of fetal superior vena cava volume blood flow as the fraction of combined ventricular cardiac output at 20–40 weeks of gestational age (GA).

		**Percentile**						
**GA (weeks)**	***n***	**2.5th**	**5th**	**10th**	**50th**	**90th**	**95th**	**97.5th**
20	16	3.63	4.19	4.95	8.87	15.91	18.78	21.67
21	29	3.63	4.18	4.93	8.82	15.78	18.60	21.46
22	25	3.62	4.18	4.92	8.79	15.68	18.47	21.30
23	30	3.63	4.18	4.93	8.77	15.61	18.38	21.18
24	17	3.64	4.20	4.94	8.77	15.57	18.32	21.10
25	27	3.67	4.22	4.96	8.79	15.57	18.31	21.07
26	26	3.70	4.25	4.99	8.83	15.60	18.33	21.09
27	26	3.73	4.29	5.04	8.88	15.66	18.39	21.14
28	24	3.78	4.34	5.09	8.96	15.76	18.49	21.25
29	18	3.83	4.40	5.16	9.06	15.89	18.63	21.39
30	33	3.90	4.47	5.24	9.17	16.05	18.81	21.59
31	24	3.97	4.55	5.33	9.31	16.25	19.03	21.83
32	17	4.05	4.64	5.44	9.47	16.49	19.30	22.12
33	24	4.15	4.75	5.55	9.65	16.77	19.61	22.46
34	25	4.25	4.86	5.68	9.85	17.08	19.96	22.85
35	25	4.37	4.99	5.83	10.09	17.44	20.37	23.30
36	16	4.49	5.14	6.00	10.34	17.84	20.82	23.81
37	16	4.64	5.30	6.18	10.63	18.29	21.33	24.38
38	24	4.79	5.47	6.38	10.95	18.79	21.90	25.01
39	17	4.96	5.66	6.60	11.30	19.35	22.53	25.72
40	2	5.15	5.88	6.84	11.68	19.96	23.23	26.50

## Discussion

### Principal Findings

The study has provided reference ranges for Q_SVC_ during the second half of pregnancy. If fetal Q_SVC_ is accepted as a representation of fetal cerebral blood flow, this study demonstrates that the blood flow to the brain increases almost 13-fold from 20 to 40 weeks of gestation. On the other hand, Q_SVC_ normalized for estimated fetal weight (mean, 49 ml/min/kg) and its fraction of fetal CCO distributed to the brachiocephalic circulation (~10%) remain stable signifying that brain development and function requires a correspondingly steady perfusion during the second half of pregnancy. This is further supported by the fact that Q_SVC_ normalized for estimated brain weight also remains stable (mean, 50 ml/min/100 g).

### Interpretation of Results

A balanced proportional distribution of fetal cardiac output to placenta/lower body and brain/upper body is essential for physiological development of fetal organ systems. Distribution of fetal cardiac output to placenta in human pregnancies has been studied relatively well-using non-invasive Doppler technique (21, 23, 24), but similar studies quantifying the physiological changes in blood supply to fetal brain and proportion of cardiac output distributed to brachio-cephalic circulation are scarce.

The present study demonstrated that the gestational age associated increase in the blood supply to the fetal brain is a result of more than 2-fold increase in the SVC diameter and almost a similar increase in the SVC blood flow velocity during 20–40 weeks. However, a corresponding increase in CCO led to a rather stable weight-indexed Q_SVC_ and weight-indexed CCO. Fetal CCO normalized to body weight is known to be relatively stable with reported mean values ranging between 400–550 ml/min/kg particularly depending on diameter measurement techniques, leading edge or inner-inner diameter (19–21). Our finding was within that range with a mean CCO of 507±12 ml/min/kg. The normalized Q_SVC_ was ~50 ml/min/kg. Considering that the Q_SVC_ is shown to increase with elapsing time after birth in uncomplicated preterm babies (from an average of 62 ml/min/kg at 5 h to 75 ml/min/kg at 12 h and 82 ml/min/kg at 24 h) (25), the value we measured in normal fetuses seems reasonable. The brachio-cephalic fraction of CCO represented by Q_SVC_ was ~10% in the second half of pregnancy, which is similar to 13% calculated from the arterial side in a previous study performed at 11–20 weeks (23).

A study performed 50 years ago that measured organ blood flow using radioactive microspheres in exteriorized human fetuses during pregnancy termination by hysterotomy at 10–20 gestational weeks, reported the mean cerebral blood flow to be 25 ml/min/100 g brain tissue and the average fraction of CCO distributed to the brain to be 14%, in 11 fetuses weighing 64–225 g (10). However, this fraction is probably smaller considering that the mean CCO was reported to be only 363 ml/min/kg, which was calculated as the sum of SVC, inferior vena cava and coronary sinus blood flow without accounting for pulmonary venous return. Furthermore, the reliability of these values is uncertain as these measurements were not performed under physiological conditions. In third trimester fetuses, we found the Q_SVC_ to be ~50 ml/min/100 g brain weight, which remained stable during 28–40 weeks. This is in line with the reported value of brachio-cephalic blood flow of 60 ml/min/100g brain weight in normotensive newborns soon after birth (26). The human fetal brain weight is about 13% of the body weight and this ratio is also relatively constant in the second half of pregnancy (11). Therefore, for an average 3.6 kg term fetus with brain weighing 468 g (13% of the body weight) the cerebral blood flow can be expected to be 234 ml/min, which is ~15% of CCO (~1,550 ml/min) at term. Considering that the Q_SVC_ includes venous return of the whole brachio-cephalic circulation rather than just the brain, our finding of 10% could be an underestimation. Moreover, as the venous return from the head, neck and upper extremities to SVC could not be examined separately, we were unable to determine relative contributions of cerebral and brachiocephalic blood flow to Q_SVC_ and any change in their proportions associated with gestational age.

The SVC blood flow is likely to be affected by several intrinsic and extrinsic factors affecting fetal heart function, such as the cardiac contractility and loading conditions, which also change with gestational age. An increased right atrial pressure could possibly reduce the Q_SVC_, but in healthy growing fetuses, the arterial pressure increases with gestation increasing brain perfusion, further aided by a reducing impedance in the brain. The increasing velocities in the SVC with gestation indicates that a sound pressure gradient drives the blood into the atrium equilibrating any physiological increase in atrial pressure. On the other hand, any abnormal increase in atrial pressure would impede flow. Q_SVC_ measurement would then be a method for assessing such a flow reduction, e.g. in case of altered cardiac function, as the reference ranges are available now.

Studies investigating blood flow distribution in near-term human fetuses using phase contrast magnetic resonance imaging (MRI) have reported equal proportions of CCO (28–29%) distributed to both the brachio-cephalic and placental circulation in late third trimester (27, 28). However, it is less likely, since the placental size is bigger in comparison to the fetal brain size (17 vs. 13% of fetal bodyweight at term), placental vascular resistance is lower as demonstrated by cerebroplacental impedance ratio during the second half of pregnancy (29, 30), and placental oxygen consumption is higher (37 ± 12 ml/min/kg) (31) compared with fetal cerebral oxygen consumption (4 ± 1.2 ml/min/kg) (32).

Proportional distribution of CCO to the brachio-cephalic circulation appears to be well-regulated and relatively stable in the fetus. It does not appear to change significantly in the second half of pregnancy as shown by our present study. The increase in blood flow to the fetal brain with advancing gestation is most likely the result of increased left ventricular output due to increased pulmonary venous return. The increase in the fraction of CCO distributed to the pulmonary circulation seen in the third trimester (19) is possibly compensated by the proportionate reduction in right ventricular output directed toward the placenta, as the fraction of fetal CCO supplying the placenta has been shown to decrease from 30 to 20% in the second half of pregnancy (21). We evaluated the relation between Q_SVC_ and umbilical vein blood flow (Q_UV_) as their ratio in the same cohort and found that the mean QS_VC_/Q_UV_ ratio increased from 0.40 at 20 weeks to 0.76 at 40 weeks of gestation (13).

### Methodological Considerations

The Q_SVC_ measured by Doppler ultrasonography is one of the best studied measures of neonatal cerebral blood flow in the neonatal period, but studies in human fetuses are scarce. The present technique of combining Doppler and 2D-imaging was used in 2012 to determine fetal Q_SVC_ during the second half of physiological pregnancies and assess the impact of fetal respiratory movement (8). We have found the reproducibility of fetal Q_SVC_ measurement to be acceptable, with an intra-observer coefficient of variation of 12.7% (95% CI 11.5–13.8) and intra-class correlation coefficient of 0.98 (95% CI 0.97–0.98) (13). Recently, Q_SVC_ measurements have been performed in fetuses (27, 28) using phase contrast cardiac MRI. A such study estimated fetal Q_SVC_ normalized for estimated fetal weight to be 137 ml/min/kg, and the SVC fraction of CCO to be 28% during 37 weeks of gestation (28). These values are much higher than the values obtained using Doppler ultrasonography in our study. However, these studies were based on a very limited number of cross-sectional observations during a small gestational age window (median 37 weeks, range 30–39 weeks). Although both techniques are non-invasive and have been shown to be feasible and promising, neither of them has been experimentally validated for the measurement of cerebral blood flow. Therefore, none of these techniques can be considered as a standard. However, in the fetus with physiologically high heart rate, the MRI is likely to underestimate the maximum velocities due to the low frame rate (20 images per cardiac cycle) compared with modern ultrasound systems with frame rates >50 images per cardiac cycle. Furthermore, the problems associated with gating, loss of signal, and noise caused by maternal breathing and fetal movements can be substantial especially in earlier gestational ages.

### Strengths and Limitations

One major strength of our study is its longitudinal design which is appropriate for studying gestational age associated changes. Moreover, the longitudinal design is more robust and efficient than a cross-sectional design as it allows construction of reference intervals with one third to half of the sample size (number of participants) with same power and precision as compared to a cross-sectional study (33). We had a sufficient number of observations per gestational week to construct reliable reference ranges, but we did not stratify our analysis by fetal sex as our sample size was not sufficient to detect an anticipated small difference in brain blood flow.

Limitations of volume blood flow measurements are well-described in the literature (34). We took measures to minimize errors. Velocity and diameter measurements of the SVC were performed at a defined position shortly above the entrance to the right atrium to reduce variability. The results of our diameter measurements are similar to that of a previously published studies (8, 18). The left and right ventricular outlet were measured respectively at the aortic and pulmonary valve levels, which are clear anatomical landmarks and our cardiac output measurements are within the range of that reported previously by other investigators (19, 35).

### Implications for Research and Clinical Practice

Q_SVC_, a proxy for cerebral perfusion, is one of the most commonly used hemodynamic parameters in newborns (7), but its clinical utility in the assessment of fetal well-being has not been assessed. We have demonstrated the feasibility of quantifying fetal SVC blood flow using Doppler ultrasonography, provided normative values that could be used in future clinical studies. Moreover, it is a relatively simple method that requires measuring blood velocity and diameter of only a single vein. The caliber of SVC is large enough in the second half of pregnancy to allow diameter measurement with good axial resolution. Q_SVC_ could be used as a surrogate measure of fetal brain blood flow as it mostly represents cerebral venous return.

Longitudinal reference percentiles established in this study have physiological significance, but could also be useful in serial evaluation and surveillance of fetuses at increased risk of perinatal complications. Low Q_SVC_ reflecting reduced cerebral blood flow has been linked to adverse neurodevelopmental outcomes in preterm neonates (36). In the fetus, increased Q_SVC_ could be a sign of brain-sparing due to intrauterine hypoxemia or increased cardiac output due to hyperdynamic circulation, whereas a low Q_SVC_ could signal inability to maintain adequate blood supply to brain due to cardiac failure or failed cerebral autoregulation. Therefore, the value of Q_SVC_ in predicting perinatal outcomes merits further investigation in clinical trials.

## Conclusion

We provide reference values of blood flow in the fetal SVC as a representation of cerebral circulation. It increases during the second half of pregnancy and constitutes roughly 10% of the fetal CCO throughout that period.

## Data Availability Statement

The raw data supporting the conclusions of this article will be made available by the authors, without undue reservation.

## Ethics Statement

The studies involving human participants were reviewed and approved by Regional Committee for Medical and Health Research Ethics –North Norway. The patients/participants provided their written informed consent to participate in this study.

## Author Contributions

MS: data collection, image analysis, data interpretation, and manuscript writing/editing. JJ: data analysis, data interpretation, and critical revision of the manuscript. LH: data collection, image analysis, data interpretation, and critical revision of the manuscript. PL: data interpretation and critical revision of the manuscript. TK: project development, data interpretation, and critical revision of the manuscript. GA: conception and design of the study, project and protocol development, project management, data collection and management, data analysis, and manuscript writing. All authors read and approved the final version.

## Conflict of Interest

The authors declare that the research was conducted in the absence of any commercial or financial relationships that could be construed as a potential conflict of interest.
